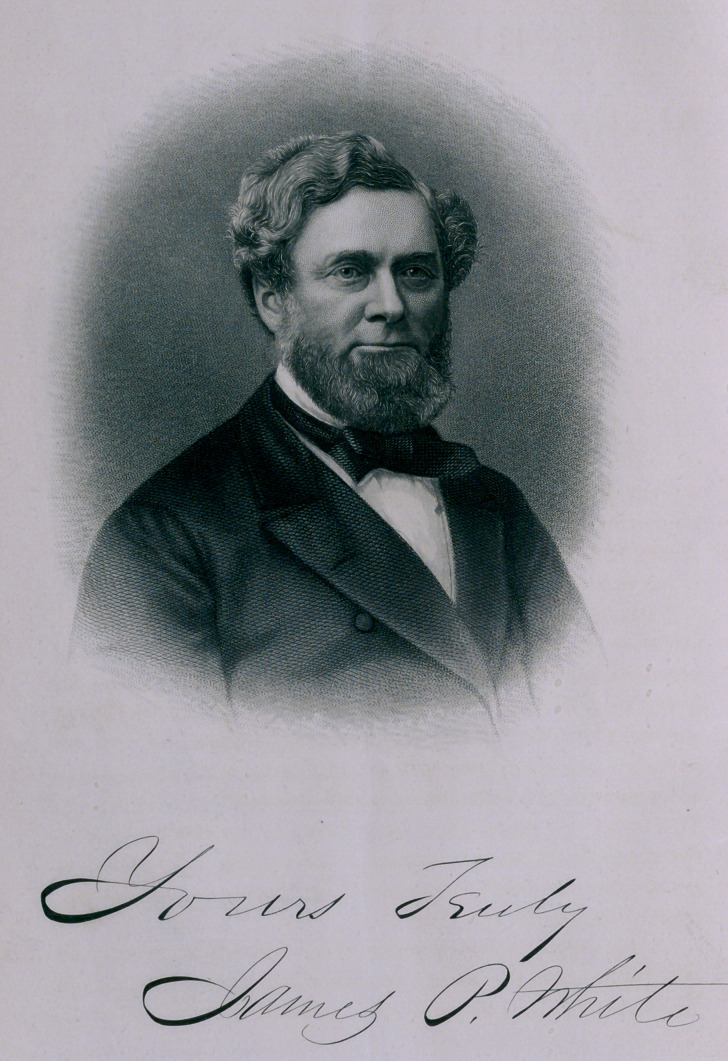# Obituary.—Professor James P. White

**Published:** 1881-11

**Authors:** 


### OBITUARY.

### PROFESSOR JAMES P. WHITE.

Within the past few weeks the medical fraternity of Buffalo and its vicinity, has sustained the loss of one of its most honored members, Dr. James Platt White, who died at his residence in this city, on Wednesday, Sept. 28th, closing a long career of assiduous devotion to his profession. He was born in Livingston County, in this State, March 14th, 1811, and therefore had, at the time of his death, exceeded man's alloted three score and ten, though his hale and vigorous appearance belied any suspicion of decay. He was essentially "of the people," his father being a farmer in by no means affluent circumstances, whose children had the spur of necessity to urge them forward on the road of life, and so similar has been the experience of many of our eminent men in America, that this early struggle with disadvantages may almost be regarded as an indispensible element of ultimate success. Certainly, Dr. White's career did not prove the fallacy of such opinion, and who can judge, how far this training in the school of adversity, served to mold the indomitable will and clear judgment, characteristic of his later years. In 1816 his father removed to Erie County, then a far western point of emigration, affording few facilities for obtaining an education, and until fifteen, young White lived at home, his sole opportunity, for acquiring knowledge being in the small schools of the village of East Hamburg. These could not, however, satisfy his ambition; he ere long, found his way to the larger academies in Genesee County, where, by teaching a portion of the time, he gained the coveted means to prosecute his studies further.

The law was his first choice as a profession, chiefly perhaps, that in the office of his uncle, Henry White, Esq., an occasion offered for legal reading; but an opportune hearing of some lectures on physiology, convinced him of the bias of his mind, and changed his whole course. All other plans were abandoned, and medicine became the mistress of his heart from that time; for its sake difficulties were overcome, and obstacles surmounted, which would have completely discouraged a less ardent lover, as such a pursuit was no easy one at that date, when the print of the red man's foot, was scarcely effaced from this section of the country. Yet the "will" discovered the "way;" in 1830 the young student gladly embraced the excellent offer of Drs. Marshall and Trowbridge of Buffalo, to enter their office. Two years later the dread disease, cholera, swept over the land, claiming hundreds as its victims, and visiting alike city and hamlet. At Black Rock—which was then disputing with Buffalo the honor of founding the future city—almost the only limit to the scourge was the number of its inhabitants. Though his brief experience scarce equipped him for the fray, White was called to this field, "fleshing his maiden sword" in this serious conflict with death, working day and night with the energy, which subsequently crowned him victor in more widely bruited contests. Two years more spent at his books, after this trial of his metal, and he graduated from Jefferson College, Philadelphia, returning immediately to Buffalo and entering into active practice. A tall, slight fellow, whose spare figure possessed few attractions, and gave no promise of that dignified presence which, in later years was one of his striking characteristics.

About this time a severe accident almost cut short his valuable life. In going to Batavia by stage—then the only means of conveyance—an upset of the coach, caused an injury to the upper part of the vertebral column; his recovery was thought at first impossible, and to the end of his days, a certain stiffness of carriage, bore witness to his marvelous escape. He married a daughter of Henry S. Penfield, of Penfield, N. Y., in 1836. She, with a nephew (his adopted son) constitute his immediate surviving family.

Buffalo physicians can glance back with pride to the succeeding ten or twenty years, for the youthful town was at that period the nursery of several practitioners, since not unknown to fame. Intimately associated with Dr. White were Drs. Flint, Hamilton and Dalton, and a union of intellects of such calibre naturally resulted in the advancement of the profession in this vicinity. To their efforts the foundation of the Medical Department of the University of Buffalo, is due—Dr. White being instrumental in obtaining the charter in 1846, and was one of its Professors from the outset.

In 1850 he began to teach midwifery, clinically, and as a pioneer in that department was met with great opposition. It was so arranged that a portion of the class then attending lectures could witness the process of delivery, and have each step clearly demonstrated. While this method of teaching was frequent enough in foreign schools, it was something uncommon or quite unknown in America, and therefore called down a torrent of abuse from some contemporaries. The secular press took up the subject, and much feeling was exhibited in the discussion for and against it. At last the matter culminated in suit for libel, which Dr. White, in the name of the people, brought against one of those opposed to him and his methods. Those were stormy days among physicians of the then provincial town, and the generation now passing away have much to relate of the word battles then fought. But, while many faults could be found with such lessons in clinical midwifery, the plan was acknowledged to be a good one, and when subsequently developed into more perfect form, was unanimously admitted to be an important step forward in the advancement of this department of science. Dr. White was also one of the founders of the Young Men's Association, and, in later years, of the Academy of Fine Arts; an able supporter and promoter of the interests of many charitable institutions, besides, including the Church Charity Foundation, the Foundling Asylum, the Eye and Ear Infirmary and others.

With the late Mr. Joseph Warren he conceived and carried into effect the establishment of the State Asylum for the Insane, remaining its President, till within a late period; was for some time President of the Medical Staff of the General Hospital; was one of the original builders of St. John's Church; nor, must it be forgotten, a large subscriber to the movement which has beautified our city with a Park, pleasure grounds and extensive avenues. In brief, whatever concerned the well-being of the community wherein he dwelt, found a ready response, a warm sympathy in the generous heart of Dr. White, and he was one, who having put his hand to the plough, looked not back, but used his unquestioned abilities to bring to a successful issue whatever was undertaken.

In 1850, and again in 1866, he visited Europe, on both occasions having more in view improvement in his loved science, than mere pleasure of travel. During his first trip he studied under the distinguished Prof. Simpson of Edinburgh—and others in Paris and Vienna; renewing, during his second visit, the valuable friendships previously formed.

The winter of 1870–71 brought him the honor of supplying the place of Prof. Elliott, as lecturer, at the Bellevue College Hospital, during the latter's illness. So acceptable did he make himself to the attending students, as well as faculty, that a handsomely engrossed testimonial was presented on his departure, as an evidence of their appreciation of the benefit derived.

Dr. White's contributions to medical literature consisted almost exclusively of reports of cases, and, addresses delivered before medical societies. They are nearly all to be found in THE BUFFALO MEDICAL JOURNAL, "The American Journal of Medical Science," "The Transactions of the American Gynecological Society" and the "Transactions of the New York State Medical Society." They date from 1845 to nearly the present time. Among them are reports of operations for ovariotomy, one case of parturition with oclusion of the vagina and os uteri, requiring section to admit of delivery; several cases of operation for chronic inversion of the uterus of varying duration; a paper upon the subject of forceps; as well as several others of lesser interest. The operation of ovariotomy he had performed more than one hundred and fifteen times.

Dr. White being a man of active habits, naturally preferred practical operations to devoting his time to the elaboration of abstruse papers, and we, therefore, find that the record shows these to have been comparatively few.

Not many men have been more earnest, unremitting workers than Dr. White, and few have reaped a more abundant harvest, for he gathered into his garners (long before his labors were ended) fame, wealth and high social position, as the well-earned reward of many years' toil. But the fruition of his hopes and aspirations did not dampen his ardor, nor render lethagic his professional ambition. His zeal showed no abatement up to the very end of his life; indeed, but for that zeal and devotion to practice, his earthly existence might have been prolonged. A short time since he was induced to perform an operation, an account of which is contained in the present number of this journal. The patient lived in a neighboring town, and he returned from the trip in a state of exhaustion, that at once excited the fears of his friends. From this he never wholly rallied, and the breaking down at last, of the grand energy, may be dated from this point. To those who fully understood his predominant trait, nothing more plainly marked the approach of the great Destroyer than the quenching of the bouyant spirit. He, who had sustained so many on the very borders of the dark valley,—almost forcing the shadows to flee away by the power of his skill,—gave himself up, and to his attending physician, Dr. Rochester,—who asked him how he felt—said simply "I am gone;" then was the golden bowl broken, the silver cord loosened forever.

The death of such a man naturally fell on the public with heavy force, calling forth universal expressions of regret.

On Sept. 29th a meeting of the Buffalo Medical Association was held, when the following resolutions were presented by Dr. Lucien Howe:

*Whereas*, Following close upon a season of universal public sorrow, it has seemed good to the Ruler of Events to deprive our city of one of her most distinguished sons, Dr. James P. White; and

*Whereas*, We, who were honored by being intimately connected with him as fellow-physicians and members of this Association, are most keenly alive to the extent of the bereavement sustained; therefore,

*Resolved*, That in the death of Dr. White the Buffalo Medical Association, the City of Buffalo, and society at large have together met with a loss which can scarcely be measured or fittingly expressed. The Association mourns one of its founders, one of its most active and eminent members, and one whose scientific zeal reflects credit upon its record. The medical profession has lost a skillful practitioner whose nerve and judgment were always to be relied upon, whose wise counsel was like a beacon-light for guidance, and whose wide reputation added a lustre to this branch of knowledge. The city of Buffalo misses an able citizen and promoter of its interest in every department, and society is deprived of a man whose Christian example will live beyond the grave, and whose energies, like his Master's, were spent in the relief of suffering humanity.

*Resolved*, That while recognizing the hand of Divine Providence, and submitting to the decrees of His unerring wisdom, we deplore the removal, of our illustrious associate while yet in the zenith of his fame and usefulness.

*Resolved*, That our grief for his loss, and the painful void caused by his absence from our midst shall be incentives to us to emulate his brilliant career; and, by following in his footsteps as physicians, in so far insures his earthly immortality.

*Resolved*, That a copy of these resolutions be transmitted to his family, and to the medical and secular press of the city.

Appropriate remarks were also made by Drs. Barker, Howe, Davidson, Rochester, Wykoff, Cronyn, Samo, Dayton, Nichell, Wetmore, Strong, Van Peyma and O'Brien. A very largely attended meeting of the Erie County Medical Society was held Sept. 30th, when addresses were made by Drs. Johnson, Ring, Folwell, Storck and Bartlett, and the following resolutions were then adopted:

*Whereas*, It has pleased God to remove from among us Dr. Jas. P. White, whose career, both professionally and otherwise, has been one of singular success; and

*Whereas,* Such as has contributed to the renown of the man, so has it to this Association, with which he has so long and so honorably been connected; and,

*Whereas,* Every member of our Society has had more or less intimate acquaintance with our departed friend, and always found him courteous, kind and instructive, how much the more is felt the great loss we have all suffered at his taking off; therefore,

*Resolved,* That we with heartfelt sorrow sympathize with the bereaved family in their misfortune, and express our great grief at the loss to the medical profession at large.

*Resolved,* That we attend the funeral in a body.

Immediately after the adjournment of this Society, the members present of the Alumni Association of the Medical Department of the University of Buffalo, were called to order. Very appropriate and fitting resolutions were offered by Dr. Barrett, which were adopted, following which extended remarks were made by him and also by Drs. Daniels and Warren.

Soon after the reassembling of the students, for their course of study at the University, they testified their deep regard for the late distinguished Dean of the Faculty, by unanimously adopting the following preamble and resolutions:

*Whereas,* It has pleased Divine Providence that, in the midst of great professional influence and usefulness, the earthly life of Dr. James P. White should, on the 28th day of September, 1881, terminate; and,

*Whereas,* His official connection with the Medical Department of the University of Buffalo, as one of its founders and for many years as Dean of the Faculty, and one of its honored and revered professors, has linked his name and memory with its history and renown; and,

*Whereas,* His professional skill and great ability as an instructor in obstetrical and gynecological science, his generous sympathy as a friend, his ripe wisdom as a counselor, have endeared him to the students, it is hereby

*Resolved,* That as students we feel that we have sustained an irreparable loss in the death of our revered and accomplished instructor and friend, Dr. James P. White.

*Resolved,* That in his death the University has lost one who ever sought its improvement and efficiency, the medical profession one of its acknowledged authorities, one of its distinguished ornaments and one of its most active and practical members.

*Resolved*, That we tender to Mrs. Dr. White our deep and sincere sympathy in her great bereavement.

GEO. STRASENBURGH,
JACOB FRANK,
CHAS. G. STRONG,
L. B. AAKER,
J. W. PUTNAM,
Committee.

There were in addition special meetings of the Board ot Managers of the Church Charity Foundation, and of the Board of Directors of the Buffalo Club, in each of which a memorial was presented, deploring the loss of their President; Dr. White holding that responsible position in both. No more convincing proof of the high esteem, in which he was held, can be adduced than the forgoing resolutions, offered by the many societies and associations of this vicinity; though, the volume would be large indeed, if we essayed to put on record the eulogies of all those who leaned on him for advice, valued him as a friend, or felt his importance as a public-spirited citizen.

## Figures and Tables

**Figure f1:**